# Treatment of Second Stage of Alveolar Abscess

**Published:** 1890-08

**Authors:** George L. Field

**Affiliations:** Detroit


Dr. George L. Field, of Detroit, read a paper on

### The Treatment of Second Stage of Alveolar Abscess.

Some weeks since I was quietly but most *emphatically* informed by the presiding officer of this association that I had been selected, elected, set apart, or whatever you may choose to call it, to write a paper npon "The Second Stage and Treatment of Alveolar Abscess," and I, in quite as emphatic a manner replied, that for many reasons too numerous to mention, I could not comply with the mandate issued. The want of time was the principal objection then in my mind, but there was one other probably quite as strong, the fact that I was quite positive that I had nothing new to offer in this field of dental science. As I felt that the subject of alveolar abscess had been so thoroughly gone over time and time again, discussed at our meetings, and spread upon paper for general circulation by those who carried wiser heads upon their shoulders than the one I had been "toting" upon mine for the last half century, that it seemed a foolish, if not reckless task for me to attempt to say more. But a second attack upon my battlements made by this same persistent officer soon followed. He said so many pretty things to me, so gently and deftly smoothed down the feathers that I felt began to stick up in every direction as he kept getting in his work upon my defences, intimating that I was one of the "*old stagers*" whom the "*boys*" would like to hear peep a last peep or two, as I gently but surely drifted into that stage of life known as the "sere and yellow leaf," that I began to melt; all these pretty words and pleasant blandishments I must admit had their weight, and I said to myself, well, any thing to oblige Benson. Then I sat down to think over my theme, "The Second Stage of Alveolar Abscess and its Treatment." And I am free to admit that the more I thought the matter over the less I could find to say. I wondered where my predecessor (No. 1) on the subject of alveolar abscess was going to end his *first* 3rd of this trinity, and where I was expected to leave it for the closing arguments of No. 3 before the court.

Alveolar abscess I have always looked upon as an effort upon the part of nature to re-establish a once normal condition. And there is one thing that should never be lost sight of, and that is, that the physician cures nothing; he can no more cure a disease than he can create a blade of grass; he may remove certain obstructions or administer certain stimulants and drugs that may assist nature in her efforts to reassert a lost supremacy, but beyond that he can not go. Life is a normal, death an abnormal condition. Alveolar abscess, as we all know, is the result of death; the death of the pulp of a tooth is nature's effort to remove that which has become an irritating cause of disturbance; the dead, or partially dead tooth, the first stage being congestion and inflammation, in which stage, by proper assistance to nature further trouble may be avoided and the abscessed stage never reached, but as these stages are not now under discussion they have no place here. What No. 1 of this alveolar abscess trinity has written upon the first treatment of the disease under consideration to read here of course I do not know, but I will take it for granted that he has opened up well the fistulous opening (that is, providing there is one), punctured the foramen, providing it was not already large enough, that he may have gotten a free passage down through the root canal, to, and through the drainage tube that nature has already tunnelled out through the process; through the same, or a similar coaxing and persuasive process that the president of this association went through to draw out these few desultory remarks from me. The case may have demanded a thoroughly heroic attack in the beginning in the way of extirpation of necrosed bone, after that the treatment he has used has no doubt been more on the persuasive order, stimulant, or sedative, or both as the case may demand, and made earnest efforts to convince nature that she was all wrong in trying to turn that poor invalid tooth out of the family household, etc., intimating that life was not all gone just because the pulp had been gathered to its fathers, but that there was still a power behind the throne in the form of a peridental membrane who would help support the family. Couldn't do it quite as well probably as the pulp, still it stood ready to do the best it could. Nature's violated law had in a measure been modified to the extent that she had already cleaned out the cesspool that smelt to heaven, began to close up the tunnel of exit by healthy granulations and allowed the offending member to quietly and gently settle down once more in the house where it was born and for many years worked for a living.

When this stage is reached the case is turned over to me. Nos. 1 and 2 are requested to tell you what I would do with it, to carry it forward to such a state of convalescence that No. 3 may take it, and with a few gentle admonitions to go and sin no more, hail him as a brother, and pass him over to the family of fellow grinders, as one snatched from the burning, a trifle weak in the lower story possibly, still strong enough that he may reasonably be expected to do his share of the household work. But where am I (No. 2) to get in any particularly different kind of work from that of my predecessor, and the more I think of it the more I do not know, unless it be that I shall represent No. 1 in a somewhat diluted form, which I might do by seeing that the pulp canal was kept clear of foreign substances and loosely filled with cotton saturated with such medicaments as the case might seem to warrant, carbolic acid, creosote, iodoform, or something of the kind, light dressings of iodine from day to day, letting time and the gradual bringing into use of the diseased member get in their work, the latter often the very best tonic that can be applied; many a tooth have I seen snatched from the forceps' grasp by simply setting it to work. Now I am through and No. 3 may come forward and close the seance by simply extending a blessing, sending in the bill, and ringing down the curtain.

Now this is about all that I know about the treatment of the "second stage of alveolar abscess." My paper may not meet the approval of our worthy president, if not, he has only himself to blame. I told him that I could give him nothing that would be worthy of consideration and probably the only thing I have done is to fully establish my reputation for truth and veracity. In brief, I do not recognize any *three stages* in the treatment of this disease, but a treatment that may begin in the heroic, and gradually taper down until there is nothing left to work upon, that is when I am successful, but unfortunately I am not, wherein I very likely differ from some of my fellow practitioners who have gotten nearer the kingdom than has been my lot, and if there be any such present these few remarks may possibly be the means of drawing them out to give us the benefit of their brighter and better experiences.

---

Dr. E C. Moore, of Detroit, read a paper on "Treatment of Third, or Chronic Stage, of Alveolar Abscess."

*Mr. Pres. and Gentlemen:*

In continuing this treatment of "alveolar abscess," as indicated in our programme, where the other two gentlemen left off. I am obliged to them for turning the case over to me for farther treatment in this particular condition or third stage, and the only stage considered by the writer to be an actual abscess in the true acceptation of the word or term, "*abscedo, and cedere,* I depart or separate from," for in this stage, to my mind, the treatment is very simple, if, indeed, the bringing about of healthy condition of affairs without medicines, can be accepted as treatment, the goal is reached more to the credit of mechanical or surgical means than to the application of actual medicines.

I am of the opinion that in almost every case of alveolar abscess the happiest results may be attained without the application of medicines unless we may construe water coming under that head. Although the writer generally uses some antiseptic or disinfectant, or in the language of Dr. George Watt, "a stink disguiser," or germ destroyer, this not so much as an element in the curative process, but for the comfort and passification of his olfactories, the treatment consists chiefly in removing the cause, or source of this outflowing fountain, the putrescing nerve, or pulp just exactly as one would remove from the ally a dead and decomposing cat or dog if in too close proximity to his residence. Its of little consequence how this is accomplished, so it is effectually accomplished, the source removed, eradicated, just a little fresh dirt thrown over the defunct animal may change the character of the air wafted through the family residence for a time, but as the elements of combustion, sun and air or heat and air, again reach the dead flesh the product of decomposition is made manifest. Just so in the treatment of the nerve canal, the dead cat must not only be thoroughly removed, but a barrier placed against its possible return, or so to speak, close up the alley. This dead matter; animal or vegetable, must be removed, and in its stead a filling of almost any indestructable substance, which must thoroughly seal the apex of the root at least. Now in the removal of this matter, and in the process of preparation for filling, there is one infallible remedy or preventive, which I am going to disclose to you as a great secret (and if it should be the only recommendation in this paper heeded the writer is content and which,if you will use,you can not fail to prevent trouble. We often hear practitioners speak of sure cures, or specifics for certain troubles, but we are apt to take it with at least a grain of allowance, but I tell you I have a sure cure or preventive, and I can demonstrate to you to a certainty its infallibility, and that is a preventive of going through the side of the root in the process of cleansing or preparing for filling. Never, never, never use a drill in this connection, its useless, its unnecessary, its bad, its senseless, its unpardonable, its criminal, and should be a States prison offense, so inexcusable is it. If this precaution or preventive is acted upon, its simply impossible to puncture the periosteum, unless there is an opening already there, but for which you can in no way be responsible, and here is the strong point in favor of this advice. If you don't use the drill, you can't be held or made responsible for any openings through the root. You are satisfied in your own conscience that you have not at any point gone through the side or bottom. With the excellent quality of broaches and canal cleaners, particularly the Donaldson, there is no excuse, and no guilty man should escape.

As I have already intimated in this short paper, its of little consequence what the canal is filled with so long as the substance is indestructable in the mouth, but both the cleansing and the filling must be thorough, only be sure of this. And to do all this it is unnecessary to wait a minute for farther treatment than has already been described in this paper. The permanent filling, so far as the canal is concerned, can be put in at once and there is the end of it, leaving the rest to the tender mercies of dame nature who only mixes a little time with her remedies.

For a filling material the writer prefers tin or oxychloride of zinc, the former being used in the straight, or single canaled teeth and is prepared from a light number of foil by shaving off with a sharp pair of foil shears into hair-like strips. One end of one of these hair-like strips is placed at the orifice of the canal to be filled and with a fine smooth broach reduced to this fineness and smoothness; by a fine oil stone and with the same squared across the end, the foil is caught by this squared end broach and carried to the extreme end of the canal, and by a slight backward and forward motion the rest of the strip is tucked in and made quite compact. This operation is repeated to the satisfaction of the operator, and in receding from the apex of the canal, and as it grows larger,larger instruments are used. It is not the intention of the writer to enter too much into minutia. All this is worked out by the operator himself.

Oxychloride of zinc is used in a class or kind of root filling where the canal is very small or difficult of access, or the canal can only be entered by using a curved instrument. A very fine broach, prepared as above described, only smaller, is used to work this cream-like mixture of oxychloride of zinc into the finest canals. A small amount of the mixture is placed at the orifice and the fine broach punctures it and passes on to the extreme end of the canal, and then a slight motion of the instrument will cause the thin mixture to disappear into the canal, removing the instrument occasionally to expel the air from the canal to allow the oxychloride to take its place. This is preferable, in the writer's estimation, to a solution of gutta-percha and chloroform; it is more liquid-like and does not stiffen like the gutta-percha owing to the rapid evaporation of the chloroform.

After this short description, passing around some of these small instruments will give you a better idea how this kind of root filling is done, and how the little instruments are prepared.

For lack of time these papers were passed without discussion.

